# Natural Colorants for a Bio-Based Economy—Recovering a Lost Knowledge for Novel Applications of *Chrozophora tinctoria* Extracts as Paints Through a Multi-Analytical Approach

**DOI:** 10.3390/molecules30132860

**Published:** 2025-07-04

**Authors:** Imogen Cleveland, Andrew Beeby, Márcia Vieira, Fernando Pina, Paula S. Branco, Paula Nabais, Maria J. Melo

**Affiliations:** 1 LAQV-REQUIMTE, Department of Conservation and Restoration (DCR), Nova School of Science and Technology, 2829-516 Caparica, Portugal; i.cleveland@campus.fct.unl.pt (I.C.); mc.vieira@campus.fct.unl.pt (M.V.); 2 Department of Chemistry, Durham University, Durham DH1 3LE, UK; andrew.beeby@durham.ac.uk; 3 LAQV-REQUIMTE, Department of Chemistry, Nova School of Science and Technology, 2829-516 Caparica, Portugal; fp@fct.unl.pt (F.P.);; 4 Institute of Medieval Studies (IEM), NOVA University of Lisbon, Av. Prof. Gama Pinto, 1646-003 Lisboa, Portugal

**Keywords:** *Chrozophora tinctoria*, microspectrofluorimetry, organic dyes, cultural heritage

## Abstract

Natural colorants, with their sustainable origins, offer a promising alternative for various applications. Advanced studies have unveiled the remarkable properties, resilience, and durability of these ancient dyes, which our ancestors developed through sustainable material processing. This serves as a testament to the potential of sustainable solutions in our field. As part of our research, we prepared three medieval temperas using gum arabic, parchment glue, and casein glue. These tempera were explicitly designed to protect the purples obtained from *Chrozophora tinctoria* extracts. A comprehensive multi-analytical approach guides our research on natural colorants. Central to this approach is the use of molecular fluorescence by microspectrofluorimetry, a key tool in our study. By analyzing the emission and excitation spectra in the visible range, we can identify specific formulations. This method is further supported by fingerprinting techniques, including Fourier Transform Infrared Spectroscopy (FTIR) and High-Performance Liquid Chromatography with Diode Array Detection (HPLC-DAD). These are further complemented by Fiber Optics Reflectance Spectroscopy (FORS) and colorimetry. Building on our understanding of orcein purples, we have extended our research to purples derived from *Chrozophora tinctoria* extracts. Our findings reveal the unique properties of *Chrozophora tinctoria*, which can be accurately distinguished from orcein purples, highlighting the distinctiveness of each.

## 1. Introduction

### 1.1. Natural Colorants

**Figure 1:**
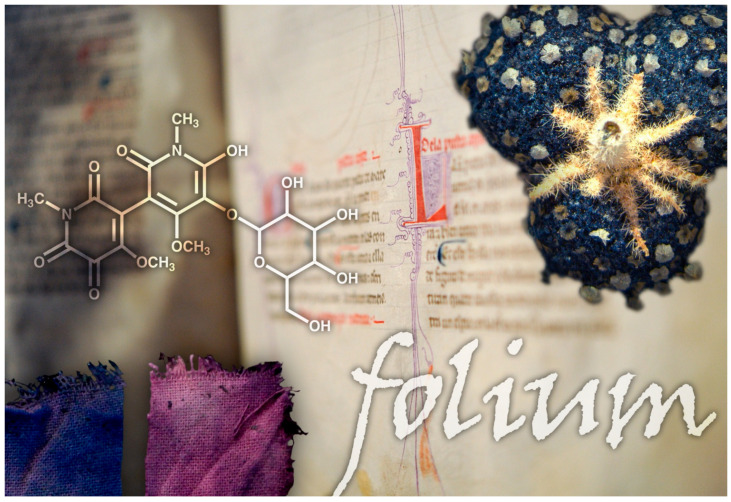
Main blue and purple for *Chrozophora tinctoria*, in clothlets, and the molecular structure for chrozophoridin blue. Prepared by designer Nuno Gonçalves.

Natural colorants can be used as sustainable sources for many applications, especially in the food and textile industry [1,2,3,4,5,6,7,8,9,10,11,12]. In Antiquity and the Middle Ages, natural dyes and their metal–ion complexes were used for textiles, manuscript illuminations, paintings, and other works of art [3,4,12], as well as in cosmetics, medicine, and food colorants [9,10,11,12,13,14,15,16,17]. Time resilience is intrinsic to these dyes, and their remarkable durability in millennial-old objects, such as Andean textiles, results from the changes introduced in the colorant formulations. This is a science of use that we inherited in the form of unique artworks [8]. This ‘science of use’ refers to the accumulated knowledge and techniques developed over centuries of practical application, which have made these colors resistant and endowed them with the highest performance that we aim to achieve [1,2,3,4,5]. We used the extracts of *Chrozophora tinctoria*, a plant found in Portugal and mentioned as a coloring material in medieval documentary sources [6]. This plant was also used for medicinal applications and to dye cheese rinds in the Netherlands from at least the 16th century to the 19th century [6,16]. Recently, we characterized the main medieval blue dye in *Chrozophora tinctoria* [6], a hermidin derivative, which we named *chrozophoridin.* Our interdisciplinary research was essential for solving its complex structure. As it turned out to be in a class of its own, this discovery was published in a high-impact journal by the Science group and widely covered by the media (Figure 1).

Contrary to other historical dyes, such as anthraquinone reds and indigo blues, the causes of the resilience of hermidin-based dyes from *Chrozophora tinctoria* still need to be understood [10,11].

### 1.2. Multidisciplinarity for the Characterization of Purple Colors Based on Chrozophora tinctoria

“Paleo-inspiration, the process of mimicking properties of specific interest observed in ancient and historical systems, is proposed for innovative chemical conception. The inspiration is gained from an advanced study of ancient materials that were often synthesized in soft chemical ways, using low energy resources, and sometimes rudimentary manufacturing equipment” [3,13,14,15,16,17,18,19,20].

In the past decade, our research on natural colorants has followed the motto described in this quotation. These advanced studies have shown ancient dyes’ remarkable properties, resilience, and durability, which our ancestors designed through sustainable materials processing. Importantly, our interdisciplinary background allowed us to assemble a multidisciplinary team of renowned experts in physical chemistry, natural products, botany, and conservation, working at the borders of their scientific domains.

A multi-analytical approach based on molecular fluorescence characterizes these formulations. This technique involves fluorescence to identify specific formulations, so it will be used as a base technique. It will be complemented by fingerprinting techniques, such as Fourier Transform Infrared Spectroscopy (microFTIR) and High-Performance Liquid Chromatography with Diode Array Detection (HPLC-DAD). Fiber-Optics Reflectance Spectroscopy (FORS) and colorimetry further support these techniques [4,7,12,20,21,22,23,24,25,26,27,28,29,30,31,32,33,34,35,36,37,38,39,40,41,42,43,44].

### 1.3. How to Prepare Purple Colors Based on Chrozophora tinctoria

The recipe for folium has been described in various manuscripts and medieval sources [5,6,7,14,18]. The plant described in these recipes is widely believed to be *Chrozophora tinctoria*, a plant native to the Mediterranean, North Africa, and parts of Asia, based on several medieval treatises that provide detailed and accurate descriptions of the fruit. The fruits are dark green, purple, or blue, depending on the time of year they are picked. The fruits consist of three conjoined, slightly lobed pods (5–8 mm diameter) with white scales, containing three seeds (Figure 1).

The key treatises that contain information on the collection and preparation of the fruits to make folium are as follows: *Theophilus on divers arts* (12th century), *Montpellier liber diversarum arcium* (14th century), and *The book on how to make all the color paints for illuminating books* (15th century) [14,15,16,17,18]. In medieval times, the solutions extracted from *C. tinctoria* were absorbed onto clothlets, dried, and then applied as paint by cutting a piece of cloth and extracting its color with the appropriate binding medium, such as gum arabic.

The practice of making watercolor clothlets was very common in the Middle Ages; therefore, in some uses of the word, turnsole became synonymous with any clothlet storing a water-based color, leading to some confusion about the nature of turnsole (or folium) and incorrectly linking it to other dyes, such as lichen-based dyes. There is documentation of the medieval preparation of folium in France in the 19th [9].

### 1.4. Purple Colors Based on Orcein Purples

During the medieval period, a variety of lichen species were used to make purple dyes, including the *Roccella* (the best-known orchil-producing genus), *Ochrolechia*, *Lecanora*, and *Varilaria* genera. All had a purple dye named orchil. Orchil has been recorded as a substitute for Tyrian purple since the 3rd century CE.30. Orchil dyes were accurately identified in medieval manuscripts [35,40].

**Figure 2:**
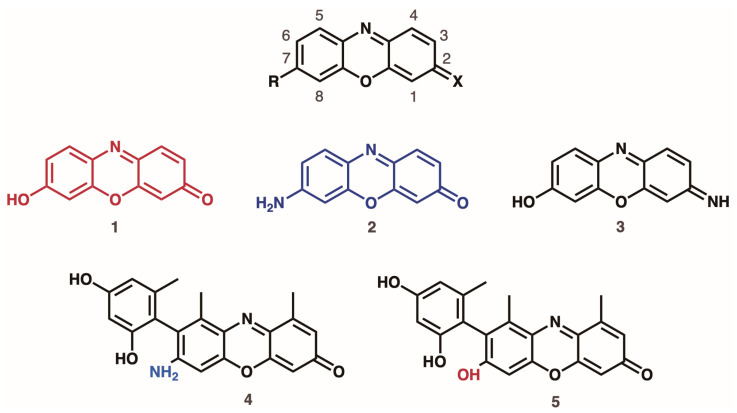
Relevant molecular structures in orcein (**1**) 7-hydroxy-2-phenoxazone, also known as hydroxy-orcein; (**2**) 7-amino-2-phenoxazone, also known as amino-orcein; (**3**) 7-amino-2-phenoxazime, also known as amino-orceimin. Relevant molecules for orcein purples, (**4**,**5**).

In 2009, the complex composition and mechanism of formation of orchil were elucidated (Figure 2) [31,32,33]. The orchil lichens contain depsides and depsidones that serve as dye precursors that vary by lichen species. After extraction, these compounds are hydrolyzed into orsellinic acid, which is then decarboxylated into the colorless compound orcinol. When exposed to ammonia, orcinol undergoes oxidation to form orcein. Orcein itself is a blend of phenoxazone derivatives, containing hydroxyorceins, aminoorceins, and aminoorceiminse.

### 1.5. Natural Colorants to a Bio-Based Economy

In summary, natural colorants were used for economic, social, and artistic purposes for millennia until synthetic dyes supplanted them in the 19th century. Nowadays, the revival of natural colors is gaining momentum, and it is necessary to revive the “savoir-faire” of dyeing with the most resilient formulations. The research and experimental development of exciting new colorants will produce novel and creative knowledge that will be freely transferred to the local communities and traded in the marketplace.

## 2. Results

In medieval times, watercolors were prepared by embedding the plant extract in a cloth, often detailed in treatises. The cloth was then protected with gum arabic. The resulting color was carefully stored in a box, a testament to the importance of preserving these historical art forms. The box was designed to shield the color from light and humidity, ensuring its longevity. We now prepare linen or cotton cloths of 4 cm by 4 cm or larger.

### 2.1. Colorimetry Through Lab* Coordinates

L*, a*, and b* are the three color coordinates for the CIELab system. L* represents the lightness, and a* and b* represent the hue. L* variations range from ‘‘light’’ or white, >0, to ‘‘dark’’ or black, <0, on the z-axis; a* from red to green; b* from yellow to blue. a* > 0 represents reds and a* < 0 greens. b* > 0 yellows and b* > 0 blues.

Each watercolor formulation was analyzed using colorimetry to determine if different binders affect the perceived color of the painted folium watercolors, as shown in Table 1 and Supplementary Material S1. All formulations exhibit hues in the pink and purple range. Each formulation was tested on four different watercolor clothlets prepared with the same method. The Lab* coordinates of watercolors with specific binders tend to group together loosely, with some notable exceptions, such as PG3 and GA3 (Figure S6). Watercolors prepared with gum arabic showed more negative b* values and more positive a* values, corresponding to a shift toward the blue and red regions of the color spectrum, respectively. Generally, watercolors formulated with casein glue, parchment glue, and glair have similar Lab* values, with the gum arabic with CaCO_3_ mixture displaying slightly more positive a* values.

### 2.2. Fiber-Optics Reflectance Spectroscopy Used on Purple Watercolor Formulations

**Figure 3:**
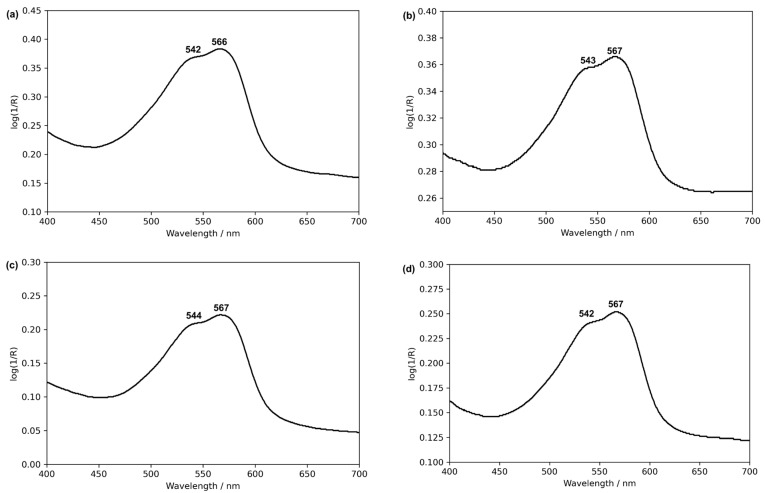
UV–VIS reflectance spectra of the following watercolors in (**a**) gum arabic, (**b**) gum arabic and CaCO_3_, (**c**) parchment glue, and (**d**) casein glue. For more details, please see Supplementary Material S1.

Each watercolor formulation was analyzed using the Fiber Optic Reflectance Spectroscopy (FORS), Figures S1–S5. The different binders have little effect on the FORS spectra with a maximum at 566–567 nm and a shoulder at 542–544 nm (Figure 3).

### 2.3. Infrared Microspectroscopy Used on Purple Watercolor Formulations

**Figure 4:**
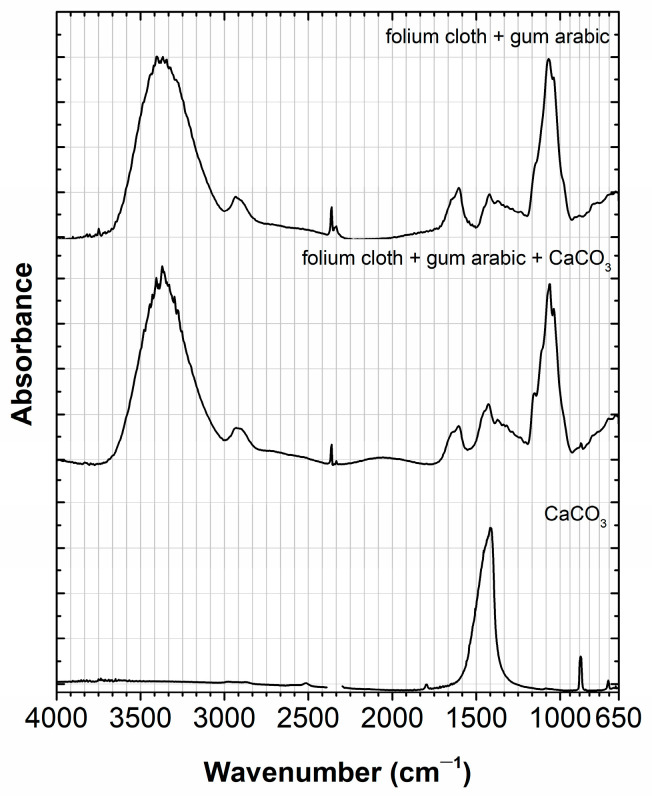
The first infrared spectrum of folium cloth in gum arabic is almost a perfect match with gum arabic (abs units 0–1). In the second spectrum (abs units 0–1.5), we can see that the quantity of calcium carbonate is very low compared to the reference CaCO_3_ (abs units 0–0.9).

**Figure 5:**
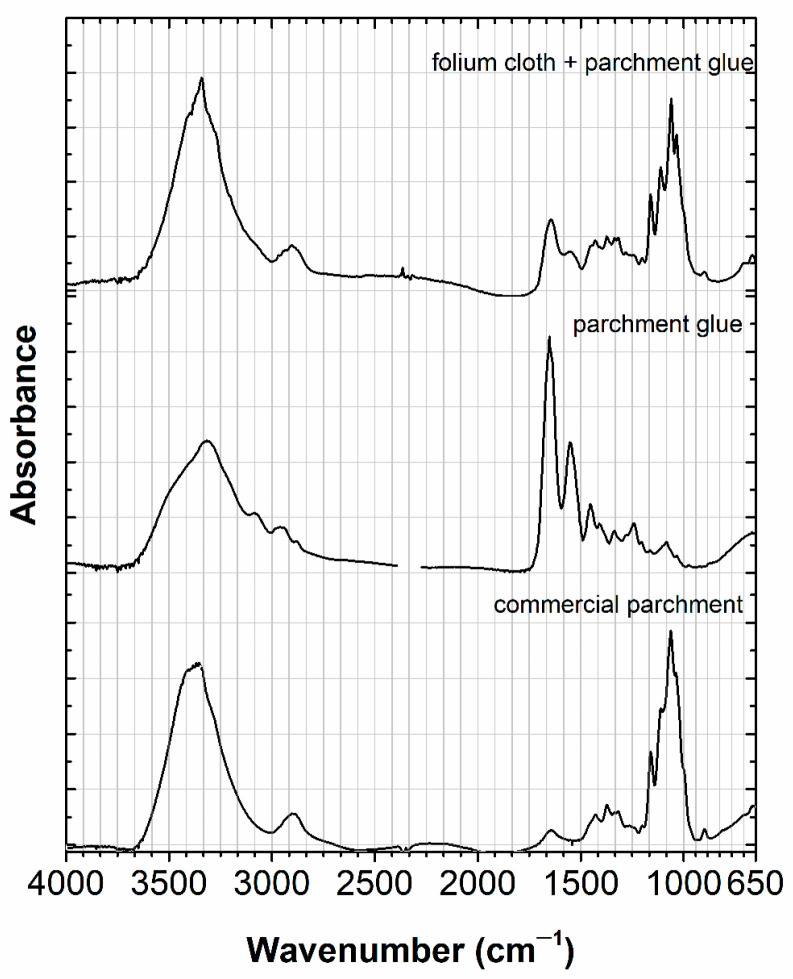
The infrared spectrum in parchment glue also includes cellulose paper, which we compared with a commercial parchment based on cellulose (abs units 0–0.7 and 0–1.5). The first spectrum is compared with a parchment glue reference (abs units 0–1.6).

**Figure 6:**
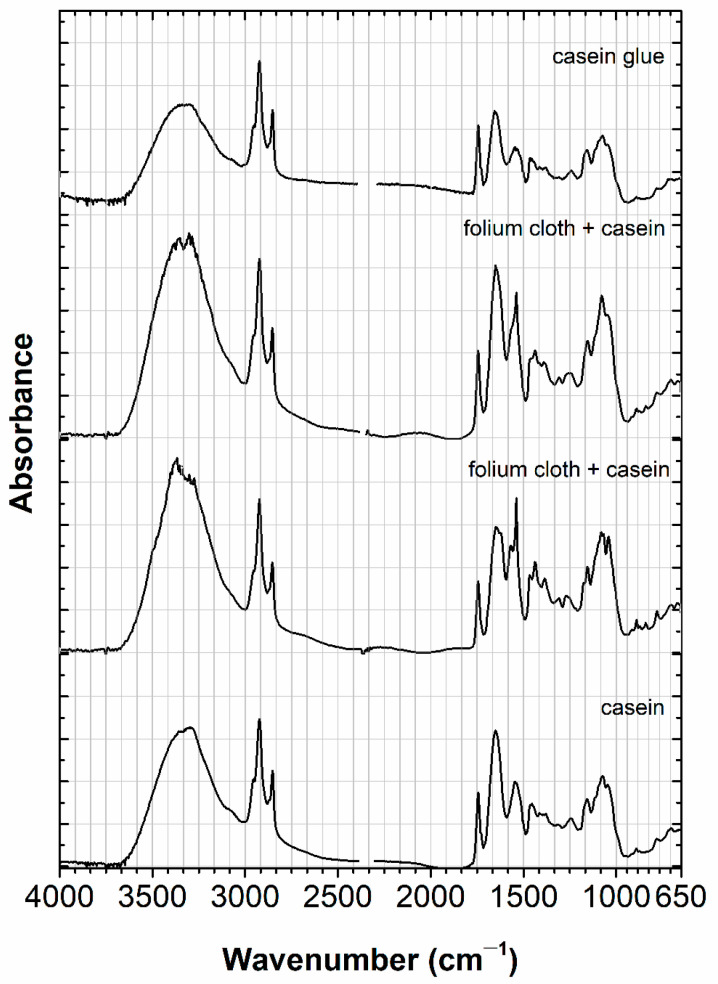
Folium extracted with casein is depicted in two infrared spectra as folium cloth + casein (abs units 0–1.2). These two infrared spectra are similar and were compared with other recipes, based on casein, prepared in the early 2000 s (abs units 0–0.2 casein glue and casein 0–0.7). For more details, please refer to the text.

The infrared spectra are depicted in Figure 4, Figure 5 and Figure 6. The fingerprint of gum arabic matches a reference for gum arabic, as shown in Figure 4. Concerning the amount of calcium carbonate, we can see its proportion is very low, with the only identified bands at 874.4 cm^−1^ and 711 cm^−1^, as shown in Figure 4. The more relevant bands for the calcium carbonate reference are at 1417 cm^−1^, 876.9 cm^−1^, and 713.3 cm^−1^. This would explain the results using colorimetry and FORS.

For the parchment glue, it was not possible to acquire spectra without interference from the cellulose paper, as shown in Figure 5. For this reason, the parchment glue is identified by its main bands at 1644 cm^−1^ and 1555 cm^−1^. The N-H and C-H bands cannot be discriminated due to the interference of the cellulose paper, in which the O-H and C-H bands dominate. When compared with a parchment glue reference, we can observe that the bands are shifted, as the main bands in this region are at 1650 cm^−1^ and 1551.5 cm^−1^.

The caseinate glue paints were applied on glass slides and as a watercolor (Figure 6). Folium extracted with casein is depicted in two infrared spectra as folium cloth + casein. These two infrared spectra are similar and were compared with other recipes, based on casein, prepared in the early 2000s. The main bands for folium cloth + casein start at the C-H region (2923 cm^−1^ and 2852/2854 cm^−1^) and continue into the fingerprint region, with the most relevant bands being 1743 cm^−1^, 1650 cm^−1^, 1539 cm^−1^, 1155 cm^−1^, 1073.5 cm^−1^, and 1046 cm^−1^. These are a very good match with the two caseins from cows.

### 2.4. Emission and Excitation Spectra by Microspectrofluorimetry

**Figure 7:**
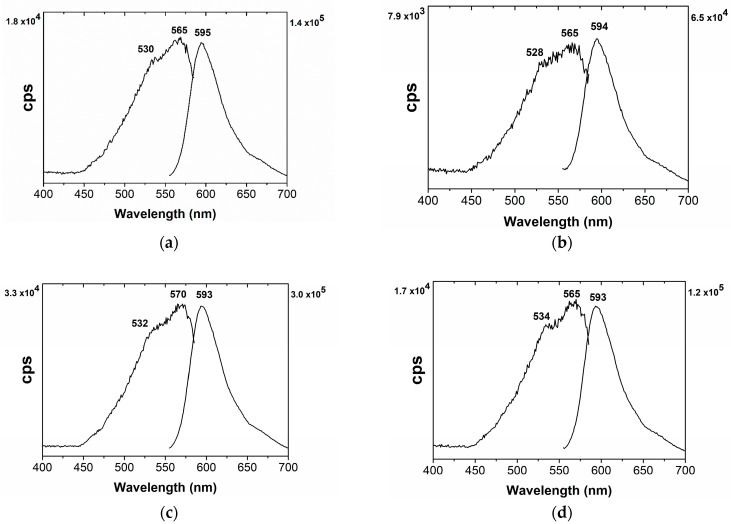
Excitation and emission spectra of the following watercolors in (**a**) gum arabic, (**b**) gum arabic and CaCO_3_, (**c**) parchment glue, and (**d**) casein glue.

Each watercolor formulation was analyzed using microspectrofluorimetry. More information on the HPLC-DAD data in Supplementary Material Section S1.4 and Table S3. Figure 7 shows a selection of the better signal-to-noise spectra. For gum arabic, the excitation spectra show a maximum at 565 nm and a shoulder at 530 and at 528 nm, when mixed with a small amount of calcium carbonate. For parchment glue, the maximum is at 570 nm, with a shoulder at 532 nm. For the casein glue, the maximum is again at 565 nm, with a shoulder at 534 nm. The emission spectra for gum arabic show a maximum at 595 nm. The maximum for parchment and casein glue is at 593 nm. However, the intensities for both excitation and emission spectra vary, as reported in Table 2.

In the infrared spectra, we can identify three different binding media, three different medieval tempera, as shown in Figure 4, Figure 5 and Figure 6. Based on the results of the emission and excitation spectra, it is also possible to distinguish gum arabic from parchment glue and casein glue. For the parchment glue, we have an excitation maximum at 570 nm and an emission maximum at 593 nm; whereas for the casein tempera, the discrimination is mainly based on the emission maximum at 593 nm. Based on microspectrofluorimetry, it is possible to discriminate these colors, as already proven in ref. [36].

## 3. Discussion

Infrared spectra, acquired using micro-Infrared spectroscopy, accurately identified the medieval tempera based on parchment glue, casein glue, and gum arabic. On the other hand, microspectrofluorimetry allowed for the discrimination of the colors prepared with *C. tinctoria*, based on their excitation and emission spectra.

In Supplementary Materials S2, we discuss the results for two types of orcein and *Chrozophora tinctoria*, which were applied on parchment. Figure S9 shows representative emission and excitation spectra for orcein purples (*Rocella tinctoria* and *Lasallia pustulata*), which present an excitation maxima at c. 585 nm and emission maxima at c. 595–598 nm [42,44]. For the orcein-dyed silk (with *Lasallia pustulata*), which was prepared by Isabella Whitworth (UK), the excitation spectrum displays a structured band, with a maximum at approximately 574 nm and a shoulder at 550 nm. The emission maximum is at 602 nm. For more details, please see reference [34]. Therefore, different supports will yield different maxima.

Orcein purples can be readily distinguished from purples based on *Chrozophora tinctoria* extracts, as shown in Figure 7 and Figure S9.

## 4. Materials and Methods

### 4.1. Preparation of Historic Paint and Ink Reconstructions

The preparation of medieval watercolors from *Chrozophora tinctoria* extracts and the preparation of colors based on medieval tempera are described in the Supplementary Materials (Sections S1.1–S1.3 and S1.5).

### 4.2. Colorimetry

For measuring color, a handheld spectrophotometer Lovibond TR 520 (Tintometer, Dortmund, Germany) with a diffused illumination system, an 8° viewing angle, and a 48 mm integrating sphere was used. The equipment was sourced from Lovibond House, Sun Rise Way. Amesbury, SP4 7GG, UK. The measuring aperture was 4 mm in diameter. Equipment calibration was performed with white and black references. Color coordinates were calculated, defining the D65 illuminant and the 10° observer. The color data are presented in the CIE-Lab 1976 system. In the Lab Cartesian system, L* refers to the relative brightness. Variations in the relative brightness range from white (L* = 100) to black (L* = 0). The (a*, b*) pair represents the hue of the object. a* ranges from negative values (green) to positive values (red). b* ranges from negative numbers (blue) to positive numbers (yellow). All colorimetric data represent the mean L*, a*, and b* values obtained from three measurements on the painted watercolor samples. The associated error corresponds to the standard deviation of these measurements.

### 4.3. Fiber-Optics Reflectance Spectroscopy

UV–VIS reflectance spectra were obtained with an Ocean Optics MAYA 2000 Pro reflectance spectrophotometer equipped with single beam optical fibers and a Hamamatsu linear silicon CCD detector collecting spectra in a 200–1060 nm spectral range. The light source was an Ocean Optics HL 2000-HP halogen lamp with a 20 W output and a 360–2400 nm spectral range. The analysis was conducted with a 35 ms integration time, 15 scans, 8 box width, and 90°/90° reflection angle to the bearing surface, with a 2 mm spot. A Spectralon^®^ white reference was used for the calibration. FORS spectra were acquired in reflectance but are presented as apparent absorbance, A’ = Log_10_(1/R).

### 4.4. Infrared Microspectroscopy

Infrared analyses were performed using a Nicolet Nexus spectrophotometer coupled to a Continuμm microscope (15 × objective) with an MCT-A detector. The spectra were collected in transmission mode, in 50 μm^2^ areas, resolution setting 8 cm^−1^, and 128 scans, using a Thermo diamond anvil compression cell. At ca 2400–2300 cm^−1^, CO_2_ absorption was removed from the acquired spectra (4000–650 cm^−1^).

### 4.5. Microspectrofluorimetry

Fluorescence excitation and emission spectra were recorded on a Jobin-Yvon/Horiba SPEX Fluorog 3-2.2 spectrofluorimeter hyphenated to an Olympus BX51 M confocal micro-scope, with spatial resolution controlled with a multiple-pinhole turret, corresponding to a minimum 2 µm and maximum 60 µm spot, with a 50 × objective. Beam splitting is achieved with standard dichroic filters mounted at 45°; they are positioned in a two-place filter holder. Standard dichroic filters of 540 and 600 nm were used to collect the emission and excitation spectra, respectively. Emission spectra were acquired by exciting at 570 nm, and excitation spectra were obtained by collecting the signal at 610 nm. Both were acquired in a 30 μm spot (pinhole 8) with the following slit settings: emission slits = 3/3/3 mm, and excitation slits = 5/3/0.8 mm. The optimization of the signal, achieved through mirror alignment in the microscope’s optical pathway, was performed for all pinhole apertures according to the manufacturer’s instructions. Spectra were collected after focusing on the sample (eye view) and then optimizing the signal intensity (detector reading). Emission and excitation spectra were acquired in the same spot whenever possible.

### 4.6. HPLC-DAD

The analysis was carried out in a ThermoScientific Vanquish^®^ HPLC-DAD system with a ThermoScientific Vanquish PDA (ThermoScientific, San Jose, CA, USA), an autosampler, and a gradient pump. The sample separations were performed in a reversed-phase column, RP-18 Nucleosil column (Macherey-Nagel, Valencienner Str. 11, 52355 Düren, Germany) with a 5 µm particle size column (250 mm × 4.6 mm), with a flow rate of 1.7 mL/min with the column at a constant temperature of 35 °C. The samples were injected via a Rheodyne injector with a 25 µL loop. The elution gradient consisted of two solvents, A: methanol and B: 0.1% (*v*/*v*) perchloric acid aqueous solution. A gradient elution program was used, with 0–2 min of isocratic 7% A, 2–8 min of linear gradient to 15% A, 8–25 min of linear gradient to 75% A, 25–27 min of linear gradient to 80% A, 27–29 min of linear gradient to 100% A, and 29–30 min of isocratic 100% A (10 min re-equilibration time). The eluted peaks were monitored at different wavelengths.

LC-MS data were obtained in the Analytical Laboratory—LAQV REQUIMTE, in Portuguese Laboratório de Análises—LAQV REQUIMTE (Ref. 10.54499/UIDB/50006/2023) at the Department of Chemistry of NOVA School of Science and Technology.

For more information on the molecular structures, see Table S3.

## 5. Conclusions

The potential of natural colorants to contribute to the sustainability of our planet is immense [1,35,36,37,38,39,40,41]. Our research, focusing on developing stable and protective formulations, as demonstrated in our study on several purples from *Chrozophora tinctoria* preserved using three different medieval tempera, is a significant step towards this optimistic future.

Our unique expertise in the field, particularly in discriminating dye formulations, is a significant contribution. We employ advanced techniques, such as microspectrofluorimetry, a method that uses a combination of fluorescence and microscopy to analyze dye formulations at a microscale, and chemometrics, a branch of chemistry that uses mathematical and statistical methods to analyze chemical data, to distinguish between organic dyes prepared in the past, a task that is crucial for the successful preservation of natural colorants [35,36]. Photodegradation studies will be applied to the paints based on *C. tinctoria* to better assess their stability.

There is an urgent and compelling need for further study of *Chrozophora tinctoria* extracts. Their potential applications remain largely unexplored, presenting an exciting and vital area for future research [40,45,46,47]. This unexplored potential underscores the urgency and importance of our work in this area.

## Figures and Tables

**Table 1:** L*, a*, and b* coordinates for the painted watercolors from clothlets 1–4 (top to bottom) in various binders (gum arabic, gum arabic with low CaCO_3_, parchment glue, and casein glue). For more details, please see Supplementary Material S1. Lab* are described in the first paragraph in Section 2.1.

Code	Binder	L*	a*	b*
GA1	gum arabic	83.24 ± 2.83	9.44 ± 1.87	−5.8 ± 1.11
GA2		81.99 ± 0.87	12.49 ± 0.63	−7.19 ± 0.37
GA3		79.08 ± 2.12	16.98 ± 1.90	−9.62 ± 1.33
GA4		84.91 ± 1.77	11.1 ± 1.55	−5.86 ± 1.18
GC1	gum arabic with CaCO_3_	82.25 ± 0.14	11.14 ± 0.17	−5.11 ± 0.08
GC2		84.41 ± 0.53	8.32 ± 0.11	−1.4 ± 0.12
GC3		85.16 ± 0.70	9.87 ± 0.27	−2.23 ± 0.28
GC4		86.85 ± 0.89	7.71 ± 0.22	−1.2 ± 0.14
PG1	parchment glue	89.95 ± 0.08	5.52 ± 0.09	−2.33 ± 0.10
PG2		90.59 ± 0.45	5.57 ± 0.57	−2.35 ± 0.41
PG3		84.46 ± 0.47	11.23 ± 0.51	−5.92 ± 0.26
PG4		89.41 ± 0.05	7.03 ± 0.17	−3.11 ± 0.11
CG1	casein glue	89.05 ± 0.29	7.14 ± 0.25	−4.11 ± 0.16
CG2		90.91 ± 0.21	4.96 ± 0.08	−1.49 ± 0.02
CG3		87.89 ± 0.28	8.33 ± 0.13	−3.79 ± 0.18
CG4		86.0 ± 0.79	10.11 ± 0.86	−5.35 ± 0.64

**Table 2:** Spectral data for painted watercolors prepared from clothlet 3 with various binders, showing the absorption, emission and excitation maxima, and the stokes shift. For more details, please see Supplementary Materials S1. GA3 in gum arabic, GC3 in gum arabic with minor amounts of CaCO_3_, PG3 in parchment glue, and CG3 in casein glue. Intensity, I/cps.

		λ_abs_/nm	Excitation		Emission		Δ $\bar{v}$ /cm^−1^
			λ_exc_/nm	I/cps	λ_em_/nm	I/cps	
GA3	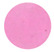	566 (*sh*542)	565 (*sh*530)	1.8 × 10^4^	595	1.4 × 10^5^	8920
GC3	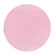	567 (*sh*543)	565 (*sh*528)	7.9 × 10^3^	594	6.5 × 10^4^	8640
PG3	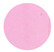	567 (*sh*544)	570 (*sh*532)	3.3 × 10^4^	593	3.0 × 10^5^	6800
CG3	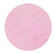	567 (*sh*542)	564 (*sh*534)	1.7 × 10^4^	593	1.2 × 10^5^	8670

## Data Availability

Data are available in the Supplementary Materials. M.J.M. can share further research data.

## Supplementary Materials

The following supporting information can be downloaded at https://www.mdpi.com/article/10.3390/molecules30132860/s1, Figure S1: Clothlets 1–12 after tempering over urine for several weeks; Figure S2: Clothlets 1–4 in gum arabic, dissolved in 1 and 2 mL of binder and applied in 1 and 2 layers, respectively; Figure S3: Clothlets 1–4 in gum arabic and with 0.015–0.017 g of CaCO_3_, dissolved in 2 mL of binder and applied in 1, 2, 3 and 4 layers (from left to right); Figure S4: Clothlets 1–4 in parchment glue, dissolved in 1 and 2 mL of binders and applied in 1 and 2 layers, respectively; Figure S5: Clothlets 1–4 in casein, dissolved in 1 and 2 mL of binders and applied in 1 and 2 layers, respectively; Figure S6: Plot of a* and b* coordinates for the painted watercolors in gum arabic (red), gum arabic with CaCO_3_ (blue), parchment glue (yellow) and casein glue (green). For more details, please see Supplementary Material S2; Figure S7: In black, the HPLC-DAD chromatogram at 300 nm of (a) extract all fruits 2 days after collection (b) extract of blue fruits 2 weeks after collection. In blue, the HPLC-DAD chromatogram at 300 nm of chrozophoridin; Figure S8: Collecting the fruits on 22 October 2024 and preparing the clothlets. For more details, see SM1.5. Figure S9: Excitation (*left*) and emission (*right*) spectra of the four dyed parchments, at *t*_0_ (unaged): (a) *Rocella tinctoria*; (b) *Lasallia pustulata*; (c) *Chrozophora tinctoria*; (d) original sample; Table S1: Lab* coordinates for the watercolors as clothlets (1–12); Table S2: Lab* coordinates for the watercolors as paints from clothlets 1–4 (top to bottom) in various binders (gum arabic, gum arabic with CaCO_3_, parchment glue, and casein) applied to paper; Table S3: Retention times and molecular ions identified from LC-MS analysis of the various fractions separated from the extract of chrozophoridin.

## References

[1] Bechtold T., Manian A.P., Pham T. (2023). Handbook of Natural Colorants.

[2] Cardon D. (2007). Natural Dyes: Sources, Tradition, Technology and Science.

[3] Bertrand L., Gervais C., Masic A., Robbiola L. (2018). Paleo-Inspired Systems: Durability, Sustainability, and Remarkable Properties. Angew. Chem..

[4] Miliani C., Monico L., Fantacci S., Romani A., Melo J., Angelin E.M., Janssens K. (2018). Recent insights into the photochemistry of artists’ pigments and dyes: Towards better understanding and prevention of colour change in works of art. Angew. Chem. Int. Ed..

[5] Melo M.J., Bechtold T., Mussak R. (2009). History of natural dyes in the ancient Mediterranean world. Handbook of Natural Colorants.

[6] Nabais P., Oliveira J., Pina F., Teixeira N., de Freitas V., Brás N.F., Clemente A., Rangel M., Silva A.M.S., Melo M.J. (2020). A 1000-year-old mystery solved: Unlocking the molecular structure for the medieval blue from *Chrozophora tinctoria*, also known as folium. Sci. Adv..

[7] Melo M.J., Nabais P., Vieira M., Araújo R., Otero V., Lopes J., Martín L. (2022). Between Past and Future: Advanced Studies of Ancient Colours to Safeguard Cultural Heritage and New Sustainable Applications. Dyes Pigments.

[8] Melo M.J., Claro A. (2010). Bright light: Microspectrofluorimetry for the characterization of lake pigments and dyes in works of art *Acc*. Chem. Res..

[9] Joly M.N. (1842). Recherches sur la Fabrication du Tournesol en Drapeaux, et sur le Principe Colorant du *Chrozophora tinctoria* (Ad. de jussieu; Croton tinctorium, Linné) Employé à cette Fabrication. Ann. Chim. Phys..

[10] Lorenz P., Hradecky M., Berger M., Bertrams J., Meyer U., Stintzing F.C. (2010). Lipophilic constituents from aerial and root parts of *Mercurialis perennis* L.. Phytochem. Anal..

[11] Lorenz P., Conrad J., Duckstein S., Kammerer D.R., Stintzing F.C. (2014). Chemistry of hermidin: Insights from extraction experiments with the main alkaloid of *Mercurialis perennis* L. tracked by GC/MS and LC/MS^n^. Helv. Chim. Acta.

[12] Melo M.J., Vieira M., Nabais P., Neves A., Pamplona M., Angelin E.M. (2024). A closer look at heritage systems from medieval colors to modern and contemporary artworks. Heritage.

[13] Clarke M. (2016). The Crafte of Lymmyng and The Maner of Steynyng: Middle English Recipes for Painters, Stainers, Scribes, and Illuminators.

[14] Clarke M. (2011). Mediaeval Painters’ Materials and Techniques: The Montpellier Liber Diversarum Arcium.

[15] Hawthorne J.G., Smith C.S. (1979). Theophilus on Divers Arts: The Foremost Medieval Treatise on Painting, Glassmaking and Metalwork.

[16] Menghini A. (2017). De Materia Medica, Il Dioscoride di Napoli.

[17] Caley E.R., Jensen W.B. (2008). The Leyden and Stockholm papyri Greco-Egyptian chemical documents from the early 4th century AD. The Leyden and Stockholm Papyri Greco-Egyptian Chemical Documents from the Early 4th Century AD.

[18] Melo M.J., Castro R., Nabais P., Vitorino T. (2018). The book on how to make all the colour paints for illuminating books: Unravelling a Portuguese Hebrew illuminators’ manual. Herit. Sci..

[19] Thompson D.V. (1932). The De Clarea of the So-Called ‘Anonymus Bernensis’.

[20] Melo M.J., Nabais P., Araújo R., Vitorino T. (2019). The Conservation of Medieval Manuscript Illuminations: A Chemical Perspective. Phys. Sci. Rev..

[21] Guineau B. (1996). Le *folium* des enlumineurs, une couleur aujourd'hui disparue. Ce que nous rapportent les textes sur l'origine et la fabrication de cette couleur, son procédé d'emmagasinage sur un morceau d'étoffe et son emploi dans l'enluminure médiévale. Identification de *folium* dans des peintures du IX^e^ s., du X^e^ s. et du début du XI^e^ s. Archéol. Médiév..

[22] Agostino A., Pellizzi E., Aceto M., Castronovo S., Saroni G., Gulmini M. (2021). On the Hierarchical Use of Colourants in a 15th Century Book of Hours. Heritage.

[23] Fiber Optics Reflectance Spectra (FORS) of Pictorial Materials in the 270–1700 nm Range. https://spectradb.ifac.cnr.it/fors/.

[24] Romani A., Clementi C., Miliani C., Favaro G. (2010). Fluorescence Spectroscopy: A Powerful Technique for the Noninvasive Characterization of Artwork. Acc. Chem. Res..

[25] Pessanha S., Manso M., Carvalho M.L. (2012). Application of spectroscopic techniques to the study of illuminated manuscripts: A survey. Spectrochim. Acta B At. Spectrosc..

[26] Vandenabeele P., Edwards H.G.M., Moens L. (2007). A Decade of Raman Spectroscopy in Art and Archeology. Chem. Rev..

[27] Bersani D., Conti C., Matousek P., Pozzi F., Vandenabeele P. (2016). Methodological Evolutions of Raman Spectroscopy in Art and Archaeology. Anal. Methods.

[28] Rosi F., Cartechini L., Sali D., Miliani C. (2019). Recent trends in the application of Fourier Transform Infrared (FT-IR) spectroscopy in Heritage Science: From micro- to non-invasive. Phys. Sci. Rev..

[29] Picollo M., Aceto M., Vitorino T. (2018). UV-VIS Spectroscopy. Phys. Sci. Rev..

[30] Aceto E.M., Calà M.J., Melo P., Nabais C., Hofmann C., Verlag B. (2020). The Vienna Genesis.

[31] Beecken H., Gottschalk E.M., Gizycki U.V., Kraemer H., Maasen D., Matthies H.G., Musso H., Rathjen C., Záhorszky U.I. (2003). Orcein and Litmus. Biotech. Histochem..

[32] Musso H., Matthies H.G. (1957). IR- und UV-Spektren Hydroxy- und Amino-Substituierter Phenoxazone. Chem. Ber..

[33] Beecken H., Gottschalk E.M., Gizycki U.V., Kraemer H., Maasen D., Matthies H.G., Musso H., Rathjen C., Záhorszky U.I. (1961). Orcein und Lackmus. Angew. Chem..

[34] Hofmann C., Vnouček J., Rabitsch S., Sonderegger J., Fiddyment S., Collins M., Quandt A., Malissa A., Uhlir K., Griesser M. (2022). The Vienna Genesis: An example of Late Antique purple parchment. Restaurator.

[35] Melo M.J., Nabais P., Guimarães M., Araújo R., Castro R., Oliveira M.C., Whitworth I. (2016). Organic Dyes in Illuminated Manuscripts: A Unique Cultural and Historic Record. Philos. Trans. R. Soc. A.

[36] Nabais P., Melo M.J., Lopes J.A., Vieira M., Castro R., Romani A. (2021). Organic Colorants Based on Lac Dye and Brazilwood Markers for a Chronology and Geography of Medieval Scriptoria: A Chemometrics Approach. Herit. Sci..

[37] Stone-Miller R. (1992). To Weave for the Sun: Ancient Andean Textiles in the Museum of Fine Arts.

[38] Paul A. (1990). Paracas Ritual Atire: Symbols of Authority in Ancient Peru.

[39] Alfaro-Saiz E., Cámara-Leret S., González-González M., Fernández-Álvarez Ó., Rodríguez-Fernández S., López-López D., Paniagua-García A.I., Acedo C., Díez-Antolínez R. (2024). The Memory of Hops: Rural Bioculture as a Collective Means of Reimagining the Future. Sustainability.

[40] Carvalho L.M. (2019). As Plantas e os Portugueses.

[41] Nabais P., Melo M.J., Clarke M., Oltrogge D., Townsend J.H., Haack-Christensen A., Stols-Witlox M. (2019). Tangled threads: Reflection on historical recipes for folium purple dye. Reflecting on Reconstructions: The Role of Sources and Performative Methods in Art Technological Studies.

[42] Córdoba de la Llave R. (2022). Interdisciplinary exploration of medieval technical manuscripts from the Iberian Peninsula. J. Mediev. Iber. Stud..

[43] Clementi C., Carlotti B., Burattini C., Pellegrino R.M., Romani A., Elisei F. (2019). Effect of hydrogen bonding interaction on the photophysics of α-aminoorcein. Spectrochim. Acta A Mol. Biomol. Spectrosc..

[44] Clementi C., Romani A., Elisei F., De Angelis F., Daus F., Nunzi F. (2021). The dependence of the spectroscopic properties of orcein dyes on solvent proticity: Insights from theory and experiments. Phys. Chem. Chem. Phys.

[45] Lorenzon V., Faccio G. (2022). Tackling Colorants Sustainability Combining Disruptive Science and Sustainable Leadership: A Review Article. Colorants.

[46] Hyttinen J., Räisänen R., Hauta-Kasari M. (2025). The BioColour Library. Color. Technol..

[47] Reazuddin Repon M., Dev M., Ashikur Rahman M., Jurkoniene S., Haji A., Abdul Alim M., Kumpikaite E. (2024). Textile dyeing using natural mordants and dyes: A review. Environ. Chem. Lett..

