# Supplementary material for: Natural Colorants for a Bio-Based Economy—Recovering a Lost Knowledge for Novel Applications of Chrozophora tinctoria Extracts as Paints Through a Multi-Analytical Approach
Source: Molecules. 2025 Jul 4;30(13):2860. doi: 10.3390/molecules30132860 (PMC12251011; doi:10.3390/molecules30132860)
Supplement: Supplementary file 1 [file molecules-30-02860-s001.zip › molecules-3680886-supplementary.pdf]

molecules

MDPI

Supplementary material 1. Preparation of the extracts from *Chrozophora tinctoria* and of the watercolors, together with the Lab* coordinates

1 *Chrozophora tinctoria*

1.1 Preparation of the watercolors

The fruits of *Chrozophora tinctoria* were collected in 22 October 2024, in Alentejo, Portugal. For more details on how the fruits were gathered, see SM1.5. The preparation of clothlets from the extracts of the fruits *C. tinctoria* was made according to recipes found in the following medieval treatises: *The book on how to make all the color paints for illuminating books* (15th century), *Montpellier liber diversarum arcium* (14th century), and *Theophilus on divers arts* (12th century) [14, 15, 18]. Twelve squares of cotton cloth were impregnated with the fresh fruits of *C. tinctoria* until they were saturated with color. The color of the clothlets varied from blue to purple. The clothlets were then left to sit over a bath of urine for several weeks to temper until they turned a purple color, Figure S1.

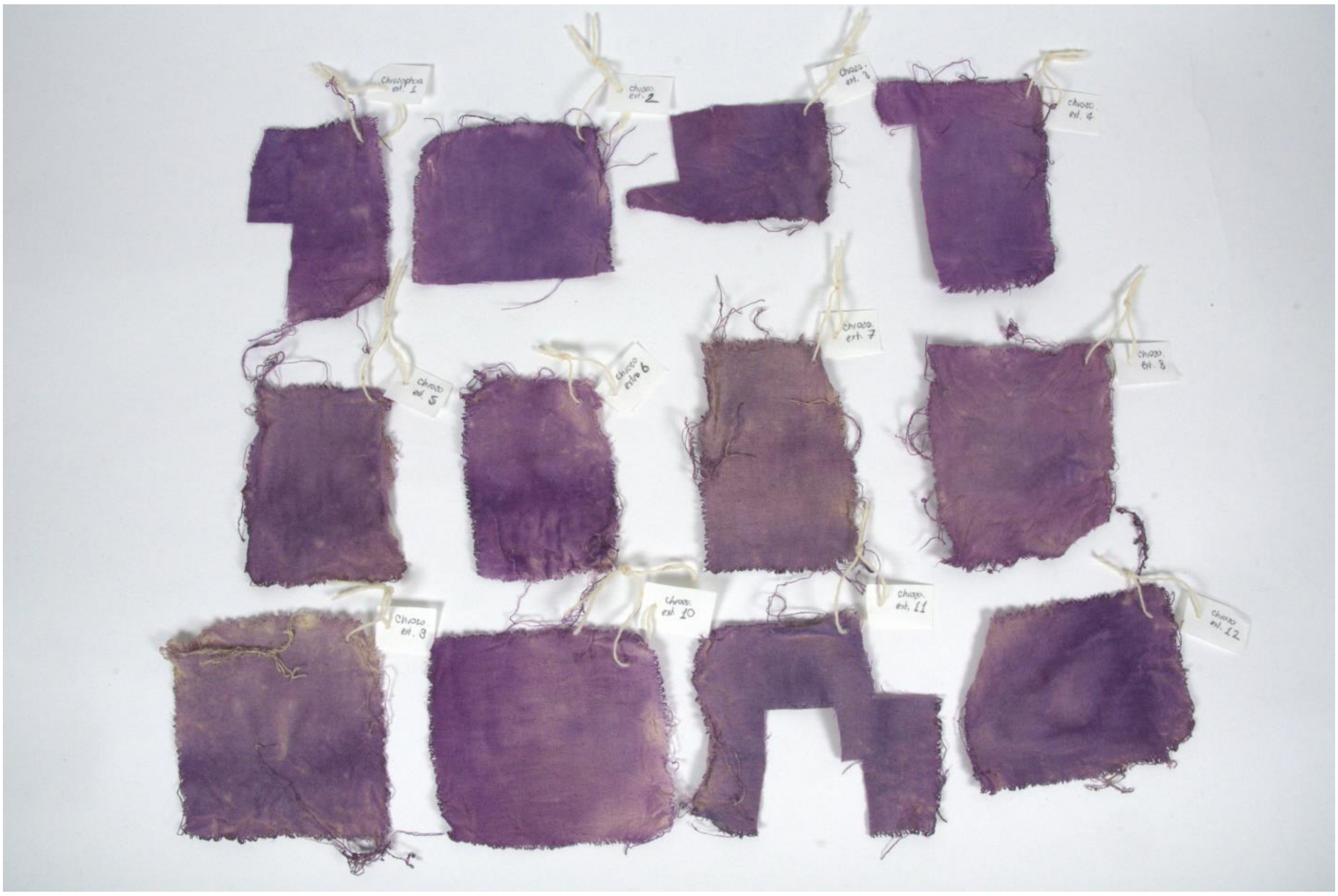

Figure S1. Clothlets 1-12 after tempering over urine for several weeks.

1.2 *Preparation of paints based on the Chrozophora tinctoria clothlets*

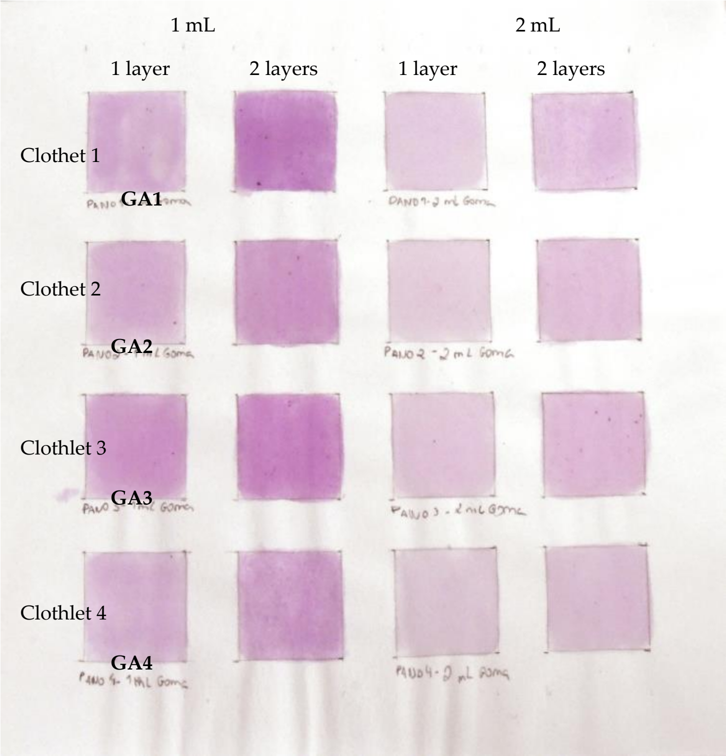

Figure S2. Clothlets 1-4 in gum arabic, dissolved in 1 and 2mL of binder and applied in 1 and 2 layers respectively.

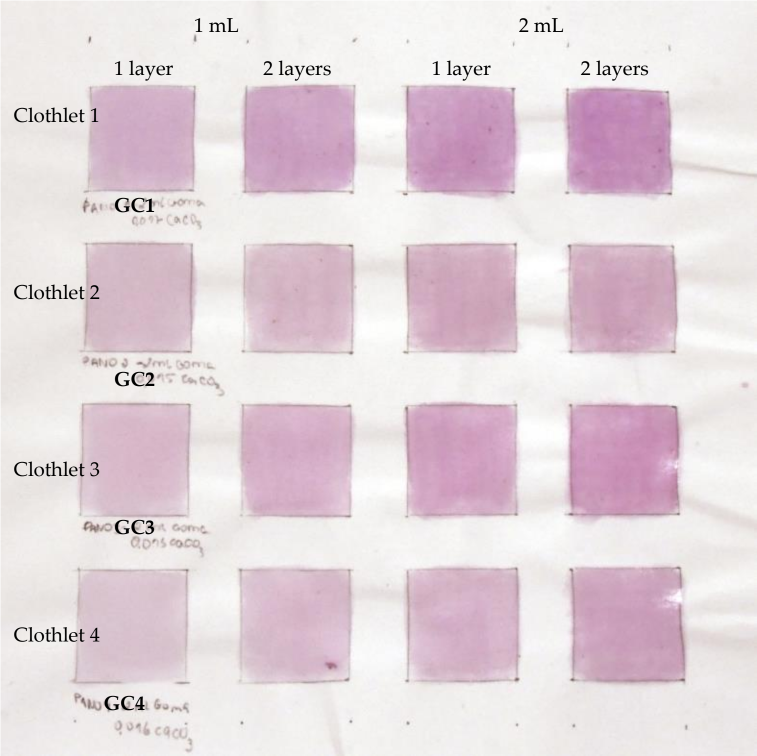

Figure S3. Clothlets 1-4 in gum arabic and with 0,015-0,017g of $CaCO_3$, dissolved in 2mL of binder and applied in 1, 2, 3 and 4 layers (from left to right).

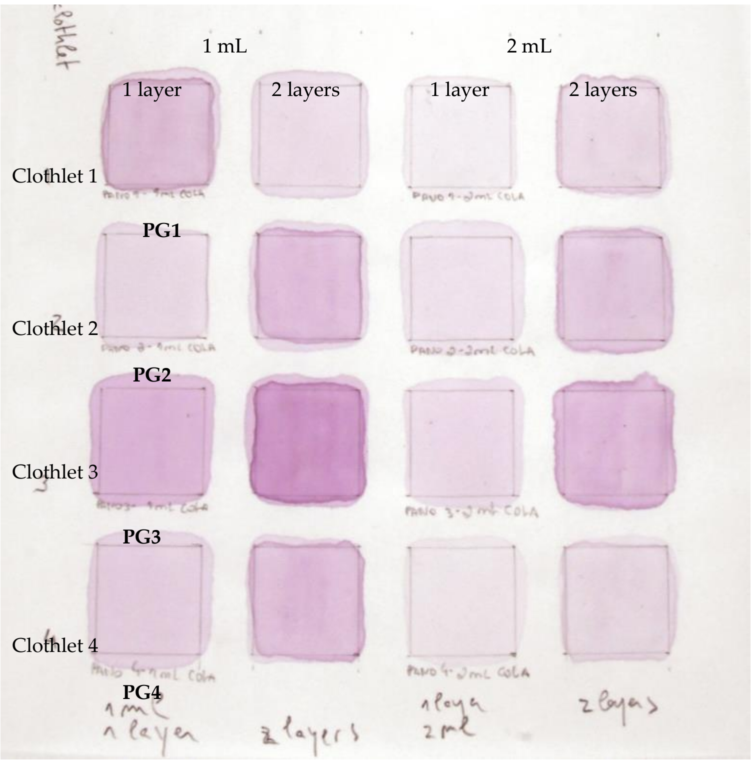

Figure S4. Clothlets 1-4 in parchment glue, dissolved in 1 and 2mL of binders and applied in 1 and 2 layers respectively.

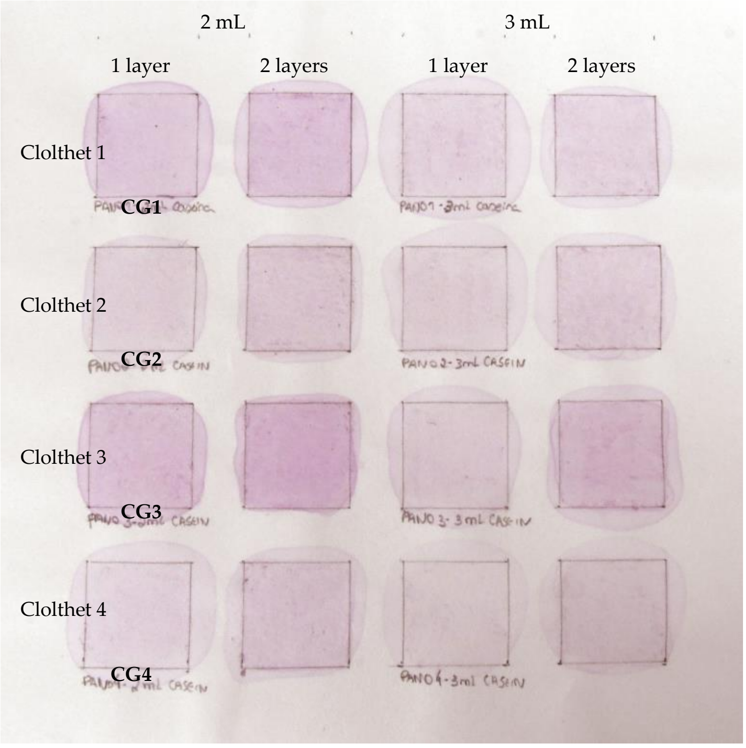

Figure S5. Clothlets 1-4 in casein, dissolved in 1 and 2mL of binders and applied in 1 and 2 layers respectively.

1.3 *Preparation of the binding media, the medieval tempera and urine bath*

1.3.1 Parchment glue

Following the recipe in The book on how to make all the colour paints for illuminating books [18]. Circa 3.5g of calf parchment (cut into small squares of 0.5cm side) was boiled in 50mL of water (80-90 °C) for 4 hours. After that, water was allowed to evaporate, until a sticky consistency was obtained.

1.3.2 Egg white

Following the recipe in *De Clarea* (11th - 12th century), pp. 14-19 [19]. Egg white was beaten with a fork and left to stand for 6 hours allowing the serum to separate. The collected serum was then used as a binder. The eggs were purchased from a local market.

1.3.3 Casein glue

Based on the recipe in *Montpellier liber diversarum arcium* (14th century), pp. 110 [14].

250 mL of a high fat commercial milk was heated (75-90 oC). Afterwards, 15 mL of vinegar was added, mixed and allowed to cool to room temperature. After filtering, 5.3 g of casein was ground with 6 mL of ash water (pH=11). Ash water was obtained by mixing ashes of Quercus sp. trees with distilled water, followed by filtration.

1.3.4 Urine bath

This recipe from *The book on how to make all the colour paints* (15th century) was reproduced with fresh fruits collected from *Chrozophora tinctoria*, near Granja-Amareleja, Portugal [15]. A linen cloth was used to collect the juice of the fruits, acquiring a green color, and these cloths were placed over wooden sticks on top of a ceramic pot full of urine. These were left there for 3 weeks, stirring the urine twice daily while turning the rags.

Table S1. Lab* coordinates for the watercolors as clothlets (1-12).

		L*	a*	b*
	Clothlet 1	43.54 ± 4.71	16.85 ± 1.57	-12.39 ± 2.11
	Clothlet 2	44.07 ± 3.28	14.39 ± 0.54	-13.19 ± 2.20
	Clothlet 3	41.3 ± 1.45	12.77 ± 2.36	-11.47 ± 0.70
	Clothlet 4	41.68 ± 4.83	14.52 ± 0.99	-11.98 ± 1.05
	Clothlet 5	48.49 ± 3.17	11.62 ± 0.69	-4.19 ± 1.45
	Clothlet 6	41.91 ± 5.73	15.78 ± 1.13	-11.63 ± 1.74
	Clothlet 7	47.73 ± 2.22	8.52 ± 0.88	-5.91 ± 0.38
	Clothlet 8	49.04 ± 0.91	12.50 ± 0.64	-7.4 ± 0.95
	Clothlet 9	52.97 ± 2.67	10.44 ± 0.63	-2.20 ± 0.10
	Clothlet 10	41.98 ± 5.10	14.04 ± 2.30	-12.34 ± 2.27
	Clothlet 11	47.51 ± 1.63	10.24 ± 0.57	-7.59 ± 0.88
	Clothlet 12	37.07 ± 2.56	13.42 ± 0.16	-12.31 ± 1.11

Table S2. Lab* coordinates for the watercolors as paints from clothlets 1- 4 (top to bottom) in various binders (gum Arabic, gum Arabic with $CaCO_3$, parchment glue and casein) applied to paper.

Code			L*	a*	b*
GA1	Watercolor in gum Arabic (1 mL)	1 layer	83.24 ± 2.83	9.44 ± 1.87	-5.8 ± 1.11
GA2			81.99 ± 0.87	12.49 ± 0.63	-7.19 ± 0.37
GA3			79.08 ± 2.12	16.98 ± 1.90	-9.62 ± 1.33
GA4			84.91 ± 1.77	11.1 ± 1.55	-5.86 ± 1.18
		2 layers	73.19 ± 1.25	17.80 ± 0.62	-12.42 ± 0.64
			77.41 ± 1.22	15.25 ± 0.14	-9.63 ± 0.62
			75.04 ± 1.69	20.82 ± 1.44	-12.09 ± 1.06
			80.32 ± 0.92	15.36 ± 0.77	-9.11 ± 0.48
	Watercolor in gum Arabic (2 mL)	1 layer	86.46 ± 0.20	8.02 ± 0.56	-3.61 ± 0.69
			87.86 ± 1.99	6.18 ± 2.15	-2.04 ± 1.58
			86.91 ± 0.08	9.45 ± 0.05	-3.81 ± 0.02
			89 ± 0.27	6.68 ± 0.25	-2.22 ± 0.17
		2 layers	85.48 ± 1.94	9.39 ± 2.21	-4.89 ± 1.40
			84.69 ± 0.03	9.83 ± 0.11	-4.13 ± 0.10
			84.50 ± 0.39	11.21 ± 0.32	-4.84 ± 0.13
			87.25 ± 0.24	8.30 ± 0.20	-3.04 ± 0.16
GC1	Watercolor in gum Arabic (1 mL) and $CaCO_3$ (0.015 g)	1 layer	82.25 ± 0.14	11.14 ± 0.17	-5.11 ± 0.08
GC2			84.41 ± 0.53	8.32 ± 0.11	-1.4 ± 0.12
GC3			85.16 ± 0.70	9.87 ± 0.27	-2.23 ± 0.28
GC4			86.85 ± 0.89	7.71 ± 0.22	-1.2 ± 0.14
		2 layers	77.99 ± 0.73	12.71 ± 0.32	-6.32 ± 0.31
			81.67 ± 1.17	8.08 ± 0.34	-1.84 ± 0.05
			80.29± 0.97	10.96 ± 0.54	-3.27 ± 0.05
			83.62 ± 0.92	8.34 ± 0.54	-2.02 ± 0.24
		3 layers	75.07 ± 0.70	15.16 ± 0.31	-7.71 ± 0.15
			78.67 ± 1.31	9.63 ± 0.81	-2.72 ± 0.12
			74.98 ± 0.41	13.07 ± 0.53	-4.06 ± 0.13
			81.96 ± 0.19	9.81 ± 0.6	-2.75 ± 0.38
		4 layers	76.23 ± 0.53	14.83 ± 0.31	-7.65 ± 0.28
			78.98 ± 1.00	10.53 ± 0.56	-2.74 ± 0.09
			77.21 ± 0.91	14.74 ± 0.48	-4.84 ± 0.15
			79.67 ± 0.44	11.45 ± 0.13	-3.62 ± 0.1
PG1	Watercolor in parchment glue (1 mL)	1 layer	89.95 ± 0.08	5.52 ± 0.09	-2.33 ± 0.10
PG2			90.59 ± 0.45	5.57 ± 0.57	-2.35 ± 0.41
PG3			84.46 ± 0.47	11.23 ± 0.51	-5.92 ± 0.26
PG4			89.41 ± 0.05	7.03 ± 0.17	-3.11 ± 0.11
		2 layers	85.02 ± 0.93	8.73 ± 0.6	-4.13 ± 0.26
			84.58 ± 0.28	9.69 ± 0.04	-5.62 ± 0.07
			77.44 ± 0.32	15.18 ± 0.18	-8.46 ± 0.22
			85.2 ± 1.07	10.16 ± 0.7	-5.1 ± 0.48

	Watercolor in parchment glue (2 mL)	1 layer	92.58 ± 0.56	3.3 ± 0.07	-0.42 ± 0.13
			89.78 ± 0.28	4.85 ± 0.13	-2.11 ± 0.06
			89.74 ± 0.13	6.57 ± 0.13	-2.79 ± 0.08
			93.33 ± 0.34	3.12 ± 0.14	-0.19 ± 0.11
		2 layers	91.03 ± 0.22	5.7 ± 0.16	-1.97 ± 0.06
			86.87 ± 1.19	7.67 ± 0.67	-4.11 ± 0.56
			85.77 ± 0.27	10.13 ± 0.32	-5.04 ± 0.23
			91.69 ± 0.34	4.83 ± 0.28	-1.08 ± 0.12
CG1	Watercolor in gum casein glue (2 mL)	1 layer	89.05 ± 0.29	7.14 ± 0.25	-4.11 ± 0.16
CG2			90.91 ± 0.21	4.96 ± 0.08	-1.49 ± 0.02
CG3			87.89 ± 0.28	8.33 ± 0.13	-3.79 ± 0.18
CG4			86.0 ± 0.79	10.11 ± 0.86	-5.35 ± 0.64
		2 layers	86.82 ± 0.56	8.67 ± 0.3	-5.05 ± 0.25
			89.46 ± 0.12	5.99 ± 0.07	-1.89 ± 0.13
			90.88 ± 0.09	5.38 ± 0.16	-1.61 ± 0.13
			89.48 ± 0.29	6.48 ± 0.37	-2.35 ± 0.19
	Watercolor in gum casein glue (3 mL)	1 layer	89.79 ± 0.59	6.11 ± 0.32	-2.39 ± 0.11
			90.48 ± 0.21	4.79 ± 0.06	-1.01 ± 0.04
			91.04 ± 0.16	5.59 ± 0.16	-1.79 ± 0.03
			93.1 ± 0.76	3.13 ± 0.61	0.43 ± 0.22
		2 layers	90.22 ± 0.57	5.63 ± 0.34	-2.15 ± 0.14
			90.18 ± 0.7	5.39 ± 0.33	-1.27 ± 0.07
			88.22 ± 0.89	7.18 ± 0.5	-2.63 ± 0.32
			91.89 ± 0.72	3.89 ± 0.29	-0.11 ± 0.1

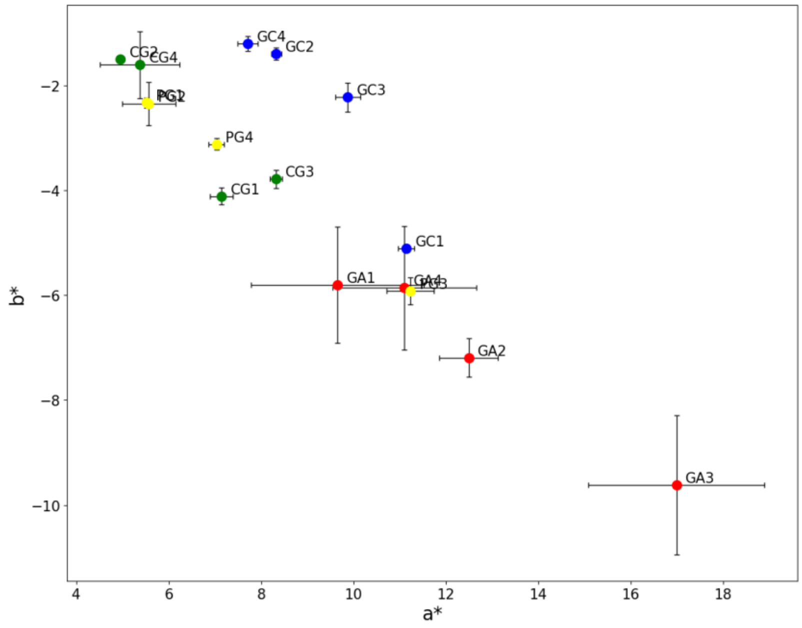

Figure S6. Plot of a* and b* coordinates for the painted watercolors in gum arabic (red), gum arabic with $CaCO_3$ (blue), parchment glue (yellow) and casein glue (green). For more details, please see Supplementary material 2.

1.4. *Identification by HPLC-DAD*

An HPLC-DAD of a known sample of chrozophoridin purified in 2021 was taken with the same acquisition method used for all other fractions purified for comparison. Then, an HPLC-DAD was taken of the crude extract from all fruits taken 2 days after collection or from selected blue fruits taken 2 weeks after collection of the fruits, Figure S7.

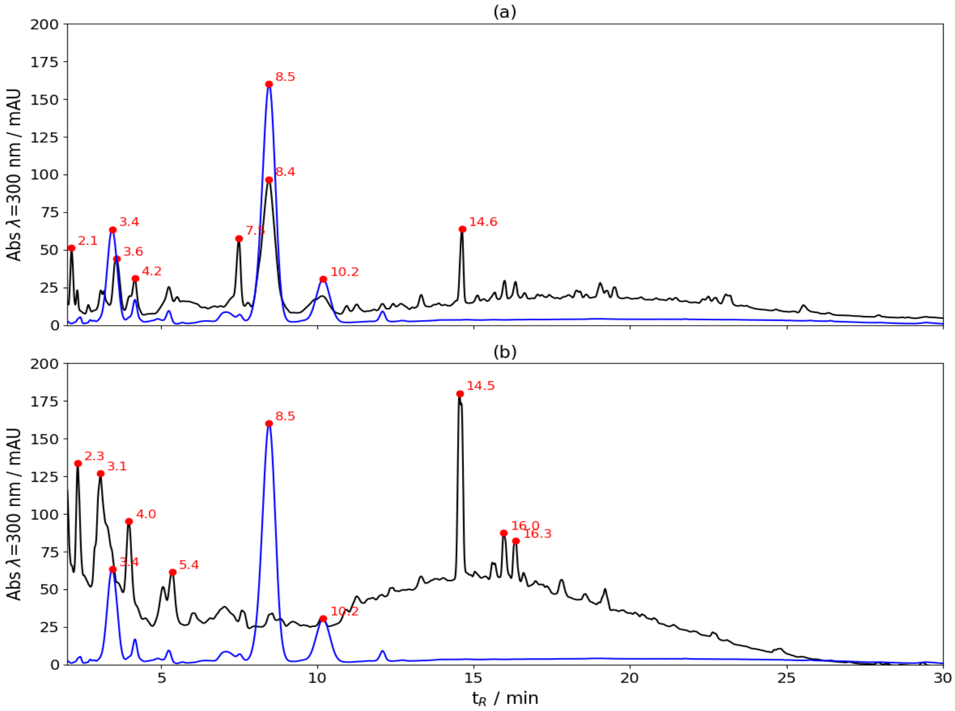

Figure S7. In black, the HPLC-DAD chromatogram at 300 nm of (a) extract all fruits 2 days after collection (b) extract of blue fruits 2 weeks after collection. In blue, the HPLC-DAD chromatogram at 300 nm of chrozophoridin.

Figure S7 of the water extract from all the fruits has a peak at t_R=8.5 min which corresponds to that of chrozophoridin (t_R=8.5 min). In figure S7b this characteristic chrozophoridin peak is not observed. Several factors may explain this observation. One possibility is that the time between the picking of the fruit and the extraction and analysis led to some degradation of chrozophoridin in the fruits. The colour and ripeness of the fruits may also influence the presence or concentration of chrozophoridin present in the fruits. Medieval sources indicate that the fruits were typically harvested in September when they were green. For more information on collecting the fruits, on 22 October 2024, please see S1.5. The blue solution of chrozophoridin was left at room temperature, and not protected on clothlets; this would explain its decomposition.

Other notable peaks include one at t_R=14.5/14.6 min, which was present in both extracts and not in the chrozophoridin sample. As well as peaks at t_R=2.0/2.3, 3.1/3.4, 4.0-4.2 and 16.0/16.3 min which were also present in both extracts.

LC-MS was subsequently employed to gain insight into the chemical structure of the compounds extracted from red *Chrozophora tinctoria*.

Table S3. Retention times and molecular ions identified from LC-MS analysis of the various fractions separated from the extract of chrozophoridin.

Retention time / min	$[M+H]^+$	$[M-H]^-$	Suggested compound
13.2	436		-
13.9	295	293	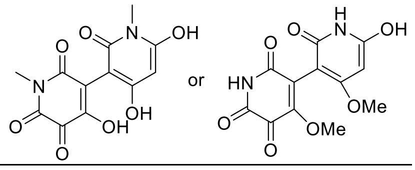
14.6	309	-	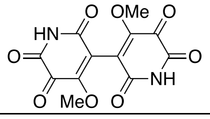
14.9	227	225	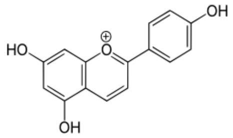
15.4	281	-	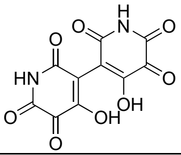
17.0	434	432	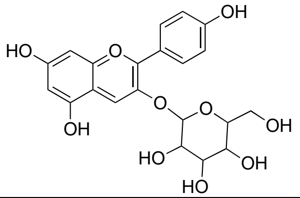
18.3	554	552	-
20.5	179	177	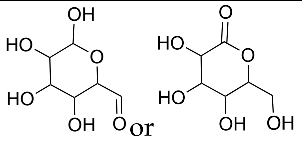
25.7	301	-	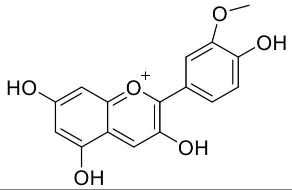

1.5 *Collecting the fruits on 22 October 2024*

The fruits of *Chrozophora tinctoria* were collected on 22 October 2024, in Granja Amareleja, Alentejo, Portugal. The green fruits were prepared as clothlets on the same day they were collected. The first two photos show where the fruits were collected and the small plant. Fruits were collected carefully from several plants. In the laboratory, at 2 pm, the green fruits were squeezed, and the third photo shows the remaining fruits used to prepare a blue color (with Milli-Q water extraction). In the two final images, the fruits were squeezed by three different persons, and were first left to dry. Then, they were placed over an urine bath as previously described in SM1.3. The clothlets gained a purple color as shown in Figure S1.

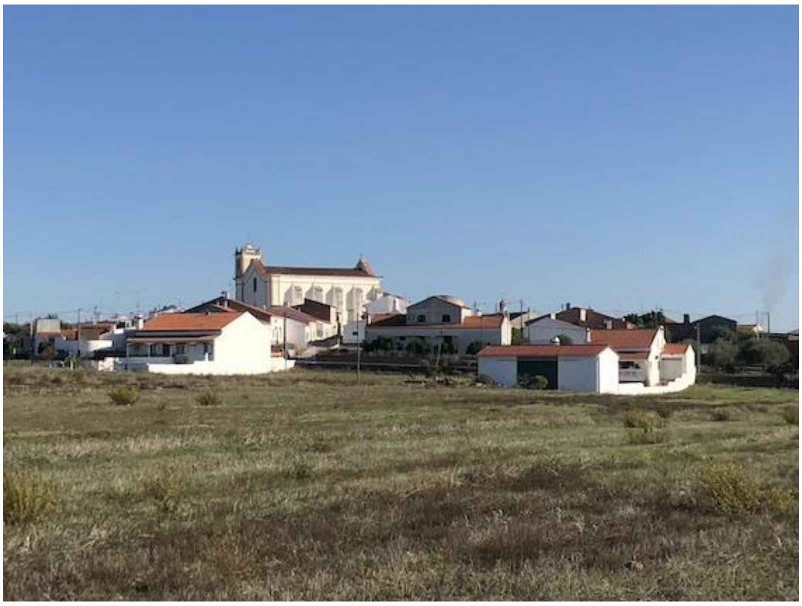

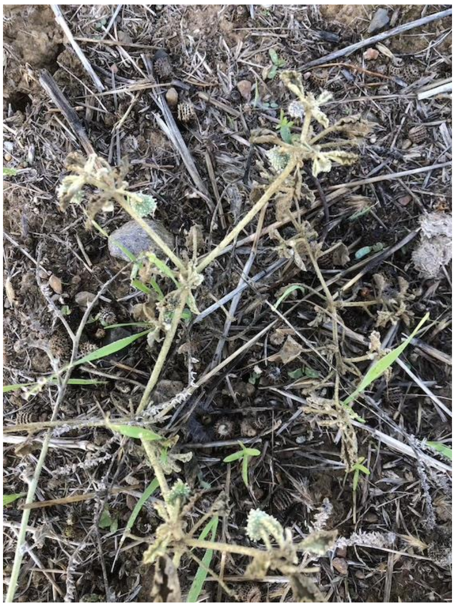

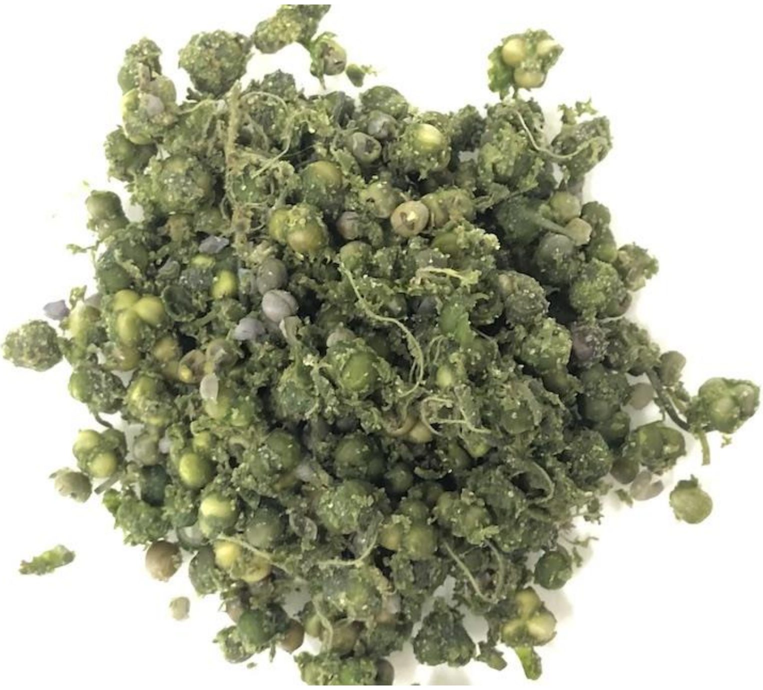

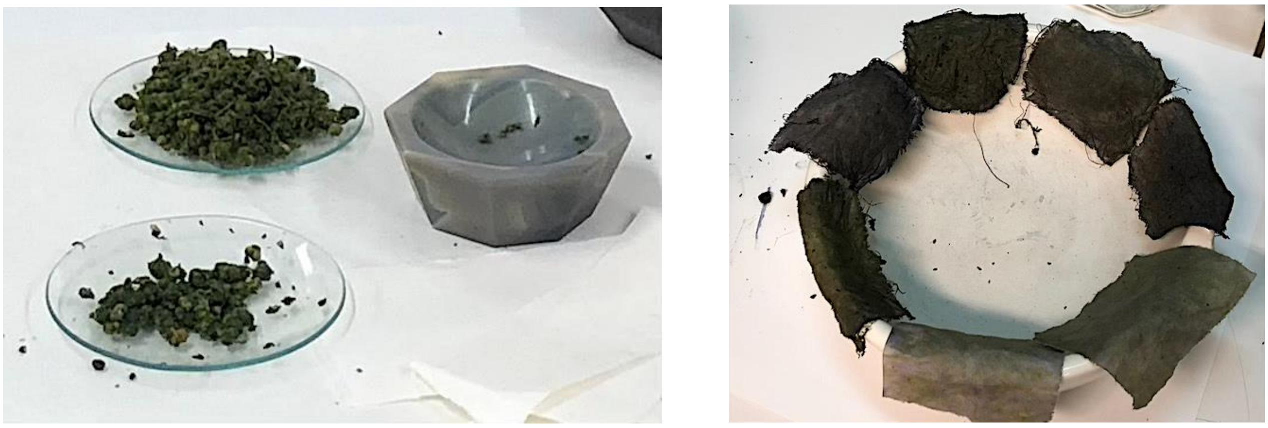

Figure S8. Collecting the fruits on 22 October 2024 and preparing the clothlets. For more details, see SM1.5.

Supplementary material 2. Spectra for orcein purples and *Chrozophora tinctoria*

Figure S7 shows representative emission and excitation spectra for the dyed reference samples, unaged. The parchment dyed with orchil dyes (*Rocella tinctoria* & *Lasallia pustulata*) presents an excitation (and absorbance) maxima c. 585nm and emission maxima at c. 595-598nm. These results agree with the previously published maxima for orcein and resorufin (hydroxyorcein) in filter paper. Spectral data for *Chrozophora tinctoria* are similar to that acquired for a Kremer blue sample. These spectra were studied during the Vienna Genesis project (2015-2018).

Orcein-dyed silk (with *Lasallia pustulata*) was prepared by Isabella Whitworth (UK),we present both the excitation and the absorption spectra for the dyed silk, for which a very good correlation is observed. The excitation spectrum displays a structured band, with a maximum at ca 574nm and a shoulder at 550nm. The emission maximum is at 602 nm. For more details please see reference 37.

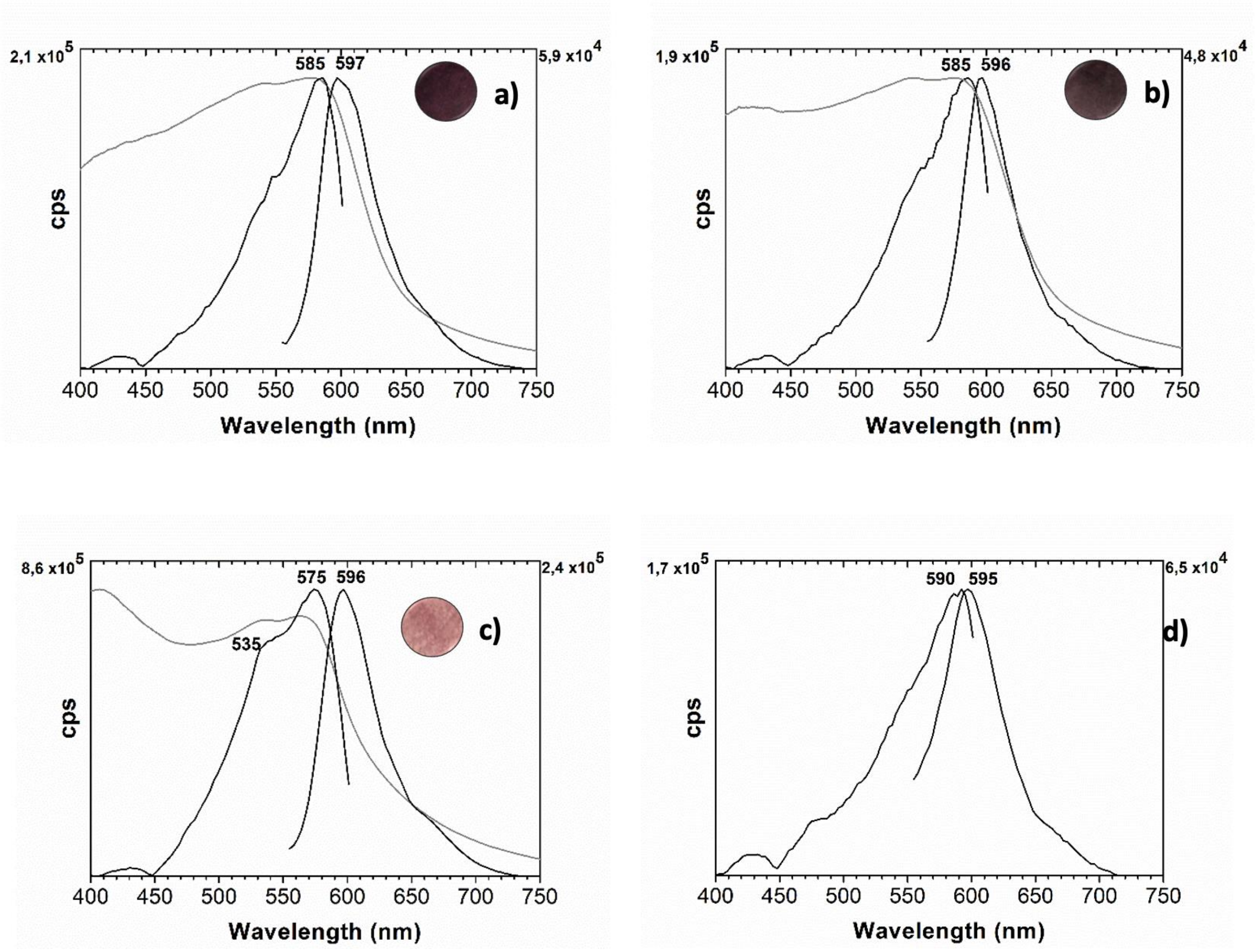

Figure S9. Excitation (*left*) and emission (*right*) spectra of the four dyed parchments, at t_0 (unaged): a) *Rocella tinctoria*; b) *Lasallia pustulata*; c) *Chrozophora tinctoria*; d) original sample.
